# Condylar volume and positional changes following a bilateral sagittal split ramus osteotomy in skeletal class II and III malocclusions

**DOI:** 10.1186/s40902-023-00408-3

**Published:** 2023-11-27

**Authors:** Chulyoung Park, Hyejin Kim, Jaeyoung Ryu, Seunggon Jung, Hong-Ju Park, Hee-Kyun Oh, Min-Suk Kook

**Affiliations:** Department of Oral and Maxillofacial Surgery, School of Dentistry, Dental Science Research Institute, Chonnam National University, 42, Jebong-ro, Dong-gu, Gwangju 61469 Republic of Korea

**Keywords:** Orthognathic surgery, Condyle, Volume change, Position change

## Abstract

**Background:**

Mandibular condyle remodeling and displacement are post-orthognathic surgery concerns that can potentially lead to occlusal issues after bilateral sagittal split ramus osteotomy. This retrospective study examined the relationship between condylar volume changes and position alterations after surgery in patients with skeletal class II and III malocclusions using cone-beam CT.

**Methods:**

The study included 16 patients (6 with Class II malocclusion, 10 with Class III malocclusion) who underwent bilateral sagittal split ramus osteotomy at Chonnam National University Hospital. Cone-beam CT data were collected at three specific time points: before surgery, immediately after surgery, and approximately 6 months post-surgery. Mandibular movement was measured using InVivoDental 5.4.6. ITK-SNAP 3.8.0 was used to assessed condylar volume changes post-surgery. Condyle positions were evaluated in four parts with RadiAnt DICOM Viewer 4.6.9. Statistical analyses were performed using the SPSS version 23.

**Results:**

Considering both Class II and III malocclusion, a 2.91% volume reduction was noted immediately and at 6 months after surgery. Both Class II and III cases demonstrated a decrease in superior joint space by -0.59 mm and medial joint space by -1.09 mm. No significant correlation was found between this process and condylar volume change.

**Conclusions:**

The mandibular condyle volume decreased, and superior-medial movement of the condyle was detected in patients with Class II and III malocclusion immediately and at 6 months after surgery with no volume-position correlation.

## Background

Orthognathic surgery is a vital surgical procedure in maxillofacial correction. The bilateral sagittal split ramus osteotomy (BSSRO), pioneered by Obwegeser and Trauner in 1955 [1], is the most utilized surgical technique for mandibular correction. This method involves cutting the mandibular ramus from above the mandibular foramen, and repositioning the mandible either in an anterior or posterior position. Subsequently, Dal Pont in 1961 and Epker in 1977 modified this surgical approach to effectively increase the contact surface between the proximal and distal segments. This modification allows for easier relocation of the mandibular condyle to the mandibular fossa, providing patients with improved masticatory function and enhanced esthetics [2, 3].

However, orthognathic surgery may give rise to certain postoperative side effects, such as remodeling and displacement of the mandibular condyle [4]. The mandibular condyle constitutes the upper part of the mandibular neck and is situated within the mandibular fossa. It exhibits an oval shape, is short anteroposteriorly, and long inward and outward. Even if there is no displacement of the bone fragment at the fracture edge, alterations in the shape and position of the mandibular condyle can lead to regression after surgery [5]. Mandibular condylar remodeling is a phenomenon defined as an adaptive physiological process, impacting the structure of the temporomandibular joint (TMJ). This irreversible process emerges from the interplay between the mechanical forces acting on the condyle and the TMJ's capacity to adapt [6]. Importantly, mandibular condyle remodeling has the potential to induce occlusal problems [7].

Various imaging techniques, including linear tomography, submentovertex radiography, lateral cephalometric radiography, and computed tomography (CT), have been employed to assess the temporomandibular joint (TMJ) [8–11]. Using conventional radiographs to accurately evaluate the TMJ can be challenging due to the superimposition of adjacent anatomical structures. Recently, cone-beam CT (CBCT) has emerged as the most valuable method for evaluating bone changes as it eliminates overlapping problems. CBCT provides the capability to evaluate both the volume and position of the mandibular condyle [12]. Additional 3D imaging programs can also aid in visualizing the positional and morphological changes of the mandibular condyle following surgical procedures.

Several previous studies have investigated mandibular condyle remodeling after orthognathic surgery using CBCT [13–15]. However, most studies have employed volume superimposition methods to evaluate alterations in the condyles and have not differentiated whether these changes were related to the condyle repositioning or the remodeling process. This retrospective study aimed to assess the relationship between condylar volume changes and alterations of the condylar position after BSSRO in patients with skeletal Class II and III malocclusions by utilizing CBCT.

## Methods

### Patients

This study included 16 patients (6 with skeletal Class II malocclusion, 6 with skeletal Class III malocclusion) who underwent only BSSRO surgery at the Department of Oral and Maxillofacial Surgery, Chonnam National University Hospital. Patients with severe facial asymmetry were excluded from the analysis. Moreover, only cases without a history of Le fort I osteotomy were included in the Class II and Class III malocclusion groups. Furthermore, individuals with a history of trauma, craniofacial syndrome, or TMD symptoms were also excluded from the study. DICOM files were collected using CBCT (DENTRI, HDXWILL Inc., Seoul, Korea) with a setting of 85 kVp and 8 mA at three specific time points: before surgery, immediately after surgery, and approximately 6 months post-surgery. This study was approved by the Institutional Review Board (IRB) of Chonnam National University Hospital (IRB No. CNUH-2023–262).

## Methods

### Measurement of mandibular movement after surgery

**Fig. 1:**
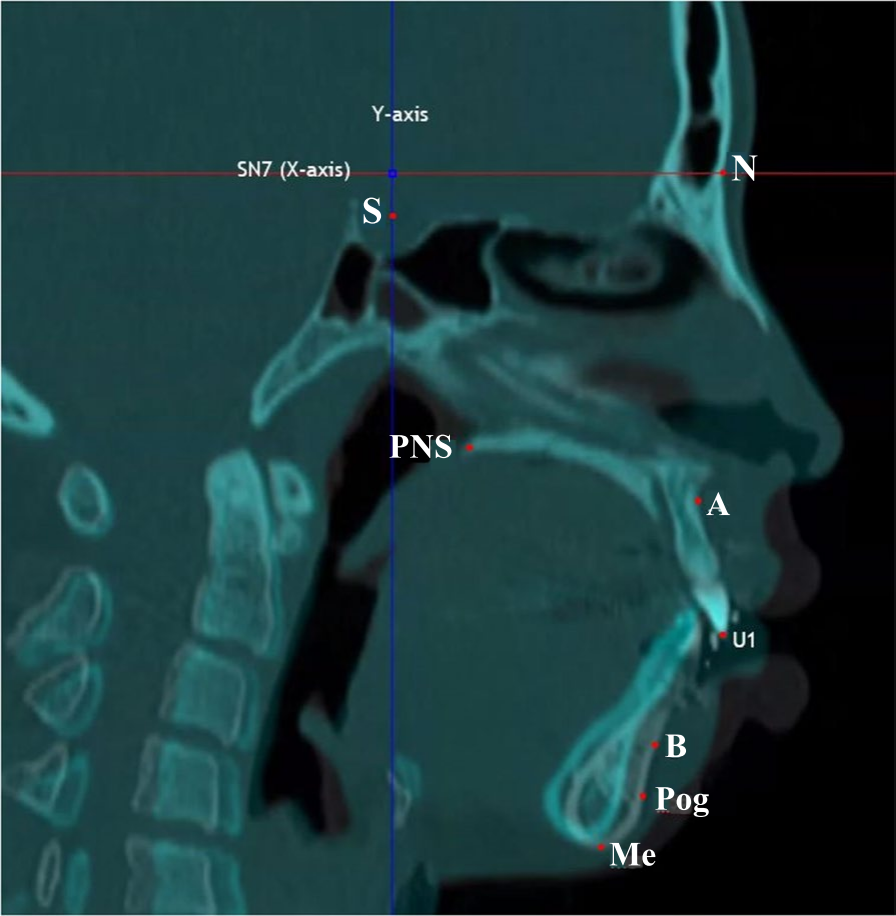
The landmarks and references for mandibular movement after surgery

The magnitude of mandibular movement was assessed by measuring the displacement of B point parallel to the Frankfort horizontal (FH) plane. This measurement was obtained after superimposing the sella turcica, nasion, A point, and PNS CT data before and after surgery, utilizing InVivoDental version 5.4.6 (Anatomage, San Jose, CA) software (Fig. 1).

### Measurement of mandibular condyle volume changes

**Fig. 2:**
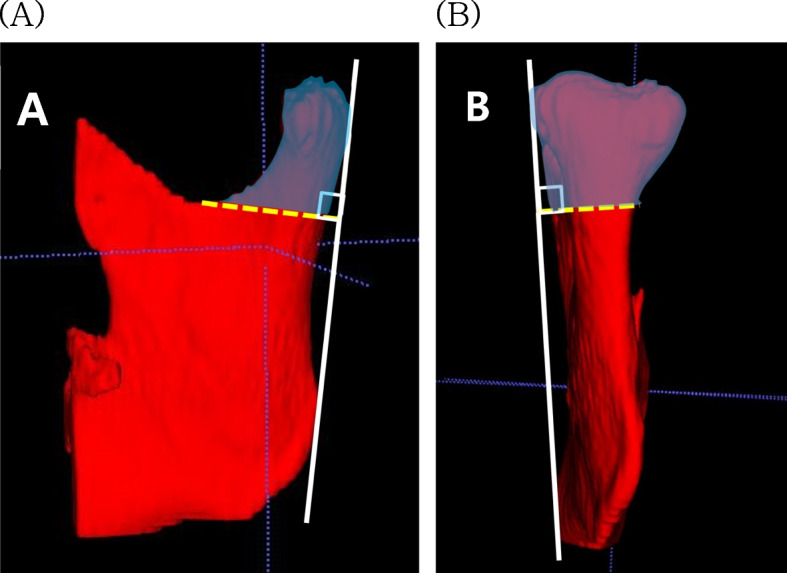
3D reconstruction of the mandibular condyle. **A** Lateral view. **B** Posterior view

Opensource software, ITK-SNAP version 4.0.1 (Cognitica, Philadelphia, PA, USA), was utilized to assess the volume change of the mandibular condyle immediately after surgery and approximately 6 months post-surgery. The three-dimensional bilateral reconstruction of the mandibular condyles was performed using CBCT data. Thresholding values were set so that the compact bone of the condyle becomes one line based on the automatic setting value of ITK-SNP. the same threshold values were maintained in the same patient immediately post-surgery and at the 6-month follow-up. The lower boundary of the mandibular condyle was defined as a plane passing through the mandibular notch and perpendicular to the tangents drawn from the posterior and lateral margins of the mandible (Fig. 2).

### Measurement of mandibular condyle position changes

**Fig. 3:**
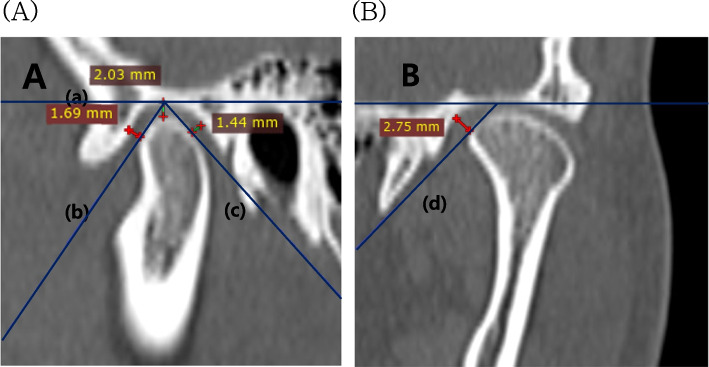
Temporomandibular condyle position measurements. A Superior (a), anterior (b), and posterior (c) joint spaces in a sagittal view of the condyle. B Medial joint space (d) in a coronal view of the condyle

The condyle positions were measured by utilizing RadiAnt DICOM Viewer version 4.6.9 (Medixant, Poznan, Poland, Central Europe), and the positions were considered from four perspectives; anterior, superior, posterior, and medial joint spaces. Considering the mandibular condyle in the sagittal plane, a tangent line (line A) parallel to the FH plane was drawn through the uppermost part of the mandibular condyle. Subsequently, two additional tangent lines were drawn, one passing through the most protruding anterior point (line B) and the other through the most protruding posterior point (line C) of the mandibular condyle. The distance from the mandibular fossa to lines B and C was measured to determine the anterior joint space (AJS) and posterior joint space (PJS), respectively. Moreover, the superior joint space (SJS) was determined as the distance from the most upwardly protruding point of the mandibular condyle to the tangent line parallel to the FH plane and passing through the highest point of the mandible. From the coronal plane of the mandibular condyle, passing through the highest point of the mandibular fossa, a tangent line (line D) was drawn to the most medial protruding point of the mandibular fossa. The distance from the mandibular fossa to line D was measured to determine the medial joint space (MJS) (Fig. 3).

### Statistical analysis

The Wilcoxon signed-rank test was conducted to assess the changes in mandibular condyle volume and position immediately after surgery and approximately 6 months post-surgery. The Mann–Whitney U test was used to compare the differences between patients with Class II malocclusion and Class III malocclusion. Additionally, Spearman's rank correlation analysis was performed to explore any potential associations between changes in condylar position and condylar volume. Spearman’s rank correlation analysis was also conducted to assess correlations between the amount of mandibular movement and changes in either mandibular condyle volume or position. The statistical analysis was carried out using the SPSS Statistics version 23 (IBM Corp., Armonk, NY, USA). A *p*-value of less than 0.05 was considered statistically significant.

## Results

The patients with skeletal Class II malocclusion (5 males and 1 female; an average age of 22.6 years) demonstrated an average of 5.49 mm of mandibular movement. In contrast, the patients with skeletal Class III malocclusion (7 males and 3 females; an average age of 22.2 years) had an average of 7.18 mm of mandibular movement of 7.18 mm.

**Fig. 4:**
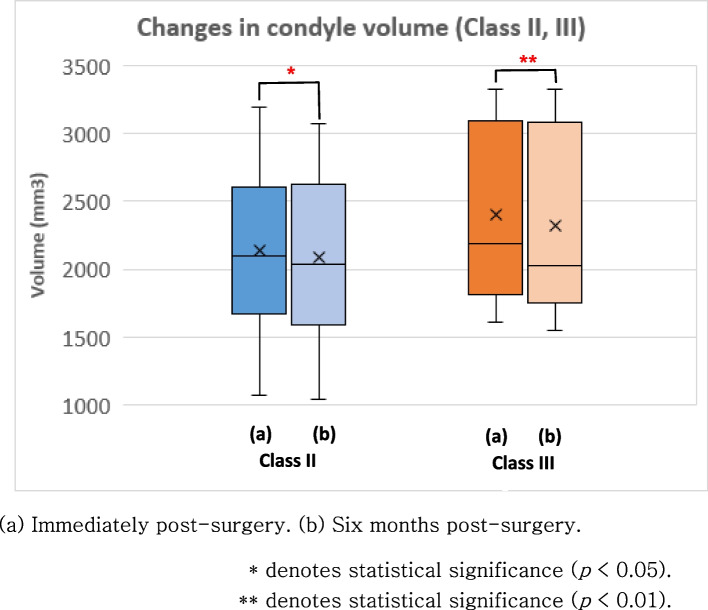
Changes in condyle volume in patients with skeletal Class II and III malocclusion, respectively

**Fig. 5:**
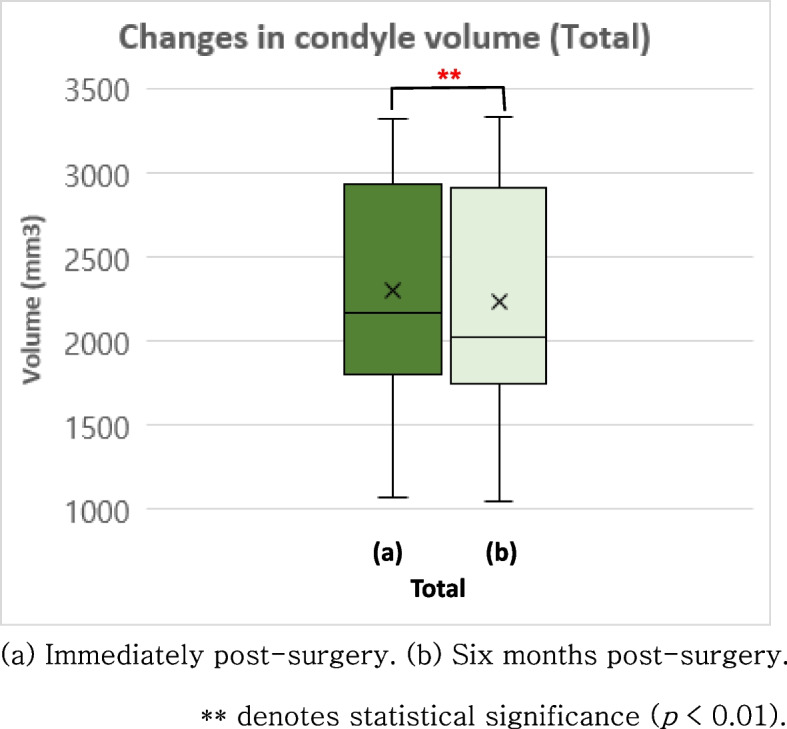
Changes in condyle volume in all patients

The Wilcoxon signed-rank test was performed to assess the volume of the mandibular condyle immediately after surgery and approximately 6 months after surgery (Fig. 4). Statistically significant reductions in mandibular condyle volume were observed among patients with Class II and III malocclusion. Specifically, a reduction of 2.78% (*p* = 0.041) was noted in those with Class II malocclusion and a reduction of 3.48% (*p* = 0.008) was observed in patients with Class III malocclusion (Fig. 4). When considering all malocclusion cases, a volume reduction of 2.91% was identified (*p* = 0.001) (Fig. 5). When considering only instances where the volume change exceeds 10%, an average reduction of 10.41% in condylar volume was observed in 8.3% of the mandibular condyles among patients with Class II malocclusion. In those with Class III malocclusion, a condylar volume reduction of 12.67% occurred in 15% of the mandibular condyles. When considering the combined results of all malocclusions, a decrease in mandibular condyle volume of 12.11% was observed in 12.5% of the mandibular condyles. In contrast, certain mandibular condyles exhibited an increase in volume, but none surpassed the threshold of 10%. The Mann–Whitney analysis revealed that a significant difference was absent in the volume change between the Class II III malocclusion groups. Moreover, the volume change between the left and right mandibular condyles was not significantly different.

**Fig. 6:**
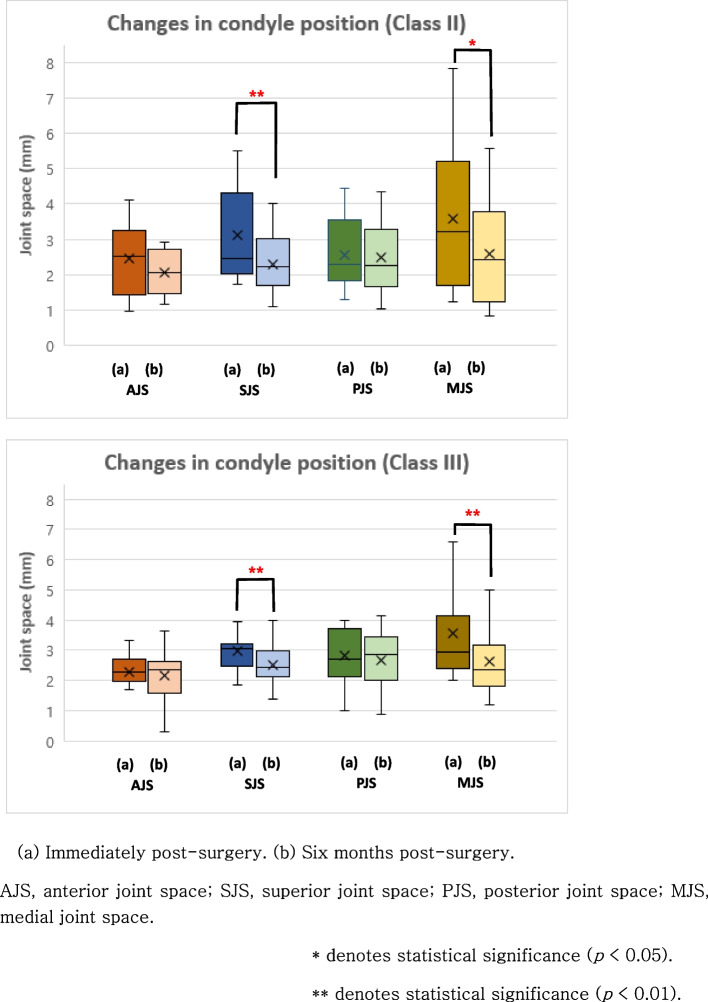
Changes in condyle position in patients with Class II and Class III malocclusion, respectively

**Fig. 7:**
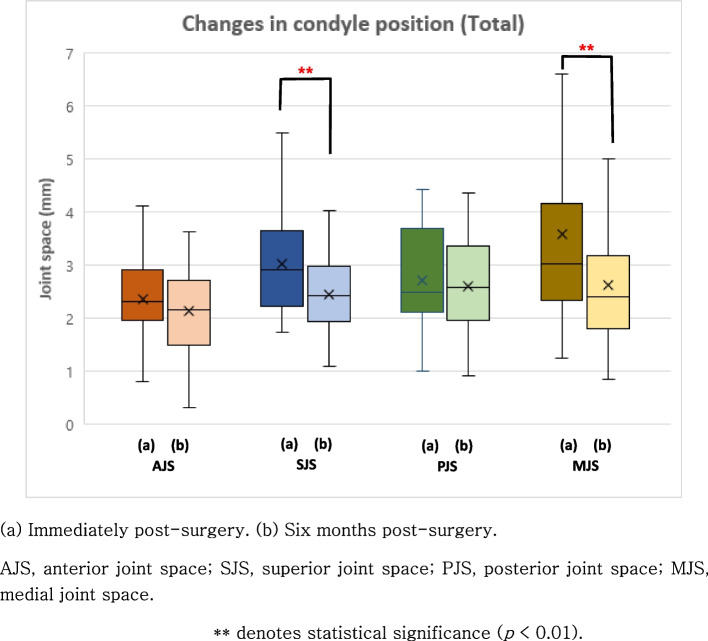
Changes in condyle position in all patients

To assess the relationship between the change in condylar volume and the positional changes of the mandibular condyle immediately after surgery and approximately 6 months post-surgery, the Wilcoxon signed-rank test was applied. In the Class II malocclusion group, there was a significant decrease in the SJS by -0.8 mm (*p* < 0.01) and a decrease in the MJS by -0.97 mm (*p* = 0.028) (Fig. 6). In the Class III malocclusion group, significant reductions were observed in the superior joint space (SJS) by -0.46 mm (*p* < 0.01) and the medial joint space (MJS) by -0.92 mm (*p* < 0.01) (Fig. 6). Combining the results from all patients, a significant decrease in the SJS by -0.59 mm (*p* < 0.01), and the MJS by -1.09 mm (*p* < 0.01) was observed (Fig. 7). The Mann–Whitney analysis indicated the absence of a statistically significant difference in the condylar position change between the two groups. Furthermore, there was no statistically significant difference in the condylar position changes between the left and right mandibular condyles.

Overall, the position of the condyle was stabilized by moving upward and inward compared to immediately after orthognathic surgery. Spearman's rank correlation analysis was performed to determine whether this process was related to the change in condylar volume. The analysis revealed there was no significant correlation (Table 1).

**Table 1:** The correlation between condylar volume changes and condylar position changes was evaluated using Spearman’s correlation coefficient

	AJS change	SJS change	PJS change	MJS change
Condylar volume changes	**0.251**	**-0.002**	**-0.58**	**-0.32**

*AJS* anterior joint space, *SJS* superior joint space, *PJS* posterior joint space, *MJS* medial joint space

In addition, Spearman's rank correlation analysis was conducted to examine the relationship between the amount of movement and the changes in the volume or position of the mandibular condyle. There was a significant correlation between the amount of mandibular advancement and AJS in the Class II malocclusion group and a significant correlation between the amount of mandibular setback and PJS in the Class III malocclusion group (Table 2).

**Table 2:** The correlation between the amount of movement and the changes in volume or position of the mandibular condyle evaluated using Spearman’s correlation coefficient

		Condylar volume change	AJS change	SJS change	PJS change	MJS change
Class II malocclusion	**mandibular advancement**	**0.269**	0.701^*^	**-0.255**	**-0.453**	**-0.276**
Class III malocclusion	**mandibular setback**	**-0.275**	**0.277**	**0.048**	0.520^*^	**0.314**

*AJS* anterior joint space, *SJS* superior joint space, *PJS* posterior joint space, *MJS* medial joint space

^*^denotes statistical significance (*p* < 0.01)

## Discussion

The primary purpose of orthognathic surgery is to correct disharmony between the maxilla and mandible with occlusion and aesthetic improvement. The fundamental prerequisite for this is the stabilization of the TMJ. In cases where the resorption of the mandibular condyle is pronounced, patients may experience occlusion issues, TMJ dysfunction, pain, facial asymmetry, mandibular retraction, and anterior open bite [7, 16]. In this regard, this paper holds significance. In this retrospective study, we compare the 2 measurements and analyze the change in condyle volume and condyle position immediately after BSSRO and approximately 6 months after surgery in patients with skeletal Class II and III malocclusion. Remodeling and displacement of the mandibular condyle in relation to orthognathic surgery are relatively common. To date, the precise mechanism and underlying cause of mandibular condyle resorption remain unclear. However, as a physiological adaptation process of the TMJ, it is postulated that resorption of the mandibular condyle begins when the external force exceeds the capacity of the joint [17]. For example, occlusal adjustments including prosthetic and orthodontic treatments may change the position of the condyle before and after treatment due to the application of different pressure to the joint than before treatment. This can lead to resorption of the mandibular condyle, but the extent is not usually significant enough to change the occlusion [18]. Patients with TMD were excluded from this study. In patients with TMD, abnormal condyle shape and volume, even if the condyle is repositioned within the glenoid fossa during surgery, the condyle's position within the glenoid fossa may misalign due to muscle reactivation, potentially leading to pathological condylar resorption [19, 20]. Many factors are causes known to increase the risk of resorption of the mandibular condyle. These include local factors, such as trauma, orthodontic treatment, and orthognathic surgery [21–25], and systemic factors, such as chronic steroid use, lupus erythematosus, and systemic sclerosis [26–28].

This study focused on orthognathic surgery as one of the potential factors contributing to the resorption of the mandibular condyle. In this study, a significant reduction in the volume of the mandibular condyle occurred in both patients with Class II and Class III malocclusion. When comparing the measurement taken immediately after BSSRO and at 6 months after surgery, the mean condyle volume immediately after surgery was 2301.12 ± 620.31 mm^3^ and 6 months after surgery was 2235.15 ± 640.49 mm^3^, showing a statistically significant decrease of 2.91%. da Silva et al. showed 3.84%, a larger average volume loss compared to the results in this study. When only a decrease of 10% or more is considered, the mean volume reduction was 12.11% in 12.5% of the mandibular condyles. da Silva et al. showed a mean volume reduction of 23.2% of the initial volume in 33.3% of the condyles.

This difference may be due to the use of data with an average follow-up period of 18 months, which is significantly longer than the 6 months in this study [29]. Moreover, although none surpassed 10%. volumetric increase in certain condyles was observed 6 months after surgery compared to immediately after surgery. Similar findings were noted in studies conducted by R.J. da Silva et al. and You Na Lim et al. These results imply that new bone formation is possible even after endochondral growth has ended in adults, and it is presumed to originate from the adaptive process through repositioning of the mandibular condyle after orthognathic surgery [29, 30]. Further research in this area is needed.

Notably, compared to immediately after surgery, the SJS and MJS showed a statistically significant decrease 6 months after surgery, suggesting that the mandibular condyle moved superiorly and medially in the mandibular glenoid fossa. da Silva et al. mentioned that changes in the position of the mandibular condyle appear to be related to other factors apart from condylar remodeling [29]. Chen et al. highlighted the possibility for intra-articular edema caused by manipulation of the mandibular proximal segment and subsequently, early-stage downward positioning of the condyle, and condylar sagging by using splints and muscle relaxants under general anesthesia [31]. In contrast, Kawamata et al. and Alder et al. reported that the displacement of the mandibular condyle is in an upward and backward direction [11, 32]. However, in these studies, the displacement of the mandibular condyle was measured using a 2-dimensional analysis method rather than a 3-dimensional method, which consequently may have affected the precision in measuring medial movements. The disparity between the findings from previous studies and the results in our study may be related to the inherent limitation in accurately quantifying the extent of inward movements.

Overall, there was no statistically significant correlation between the volume change and positional relationship of the mandibular condyle immediately after surgery and approximately 6 months after surgery, implying that they should be regarded as independent phenomena, with each may be related to distant underlying mechanisms.

Finally, a significant correlation was detected between the amount of mandibular advancement and AJS in the Class II malocclusion group, and also between the amount of mandibular setback and PJS in the Class III malocclusion group (Table 2). This result is consistent with the findings of existing studies [33, 34]. These anatomical correlations may be due to the pull of the pterygomasseteric sling caused by lengthening or stretching of the muscle fibers after BSSRO. With the amount of mandibular movement increases, there is the potential for a regressive phenomenon to occur.

This study acknowledges the limitation of the relatively small patient sample size, which may impose constraints on the generalizability and reliability of the findings. Future research efforts with larger sample sizes are required to draw more robust conclusions.

## Conclusions

This study identified a significant volume decrease and superior-medial movement of the mandibular condyle in patients with skeletal Class II and III malocclusion immediately and approximately 6 months after BSSRO surgery. However, no correlation was found between a decrease in condyle volume and positional change, implying that distinct underlying mechanisms may be involved. Additionally, patients with Class II malocclusion showed a significant correlation between mandibular advancement and AJS, while those with Class III malocclusion demonstrated a significant correlation between mandibular setback and PJS.

## Data Availability

Please contact the author for data requests.

## Abbreviations

BSSRO: Bilateral sagittal split ramus osteotomy
CBCT: Cone-beam CT
TMJ: Temporomandibular joint
FH: Frankfort horizontal plane
AJS: Anterior joint space
SJS: Superior joint space
PJS: Posterior joint space
MJS: Medial joint space
